# Synchronous occurrence of papillary thyroid carcinoma and early esophageal squamous cell carcinoma in a 45-year-old female: a case report and review of the literature

**DOI:** 10.11604/pamj.2022.42.248.36342

**Published:** 2022-08-03

**Authors:** Abdulfattah Altam, Ahmed Alsaaidi, Waleed Aljbri, Faisal Ahmed, Qasem Alyhari, Mohamed Badheeb

**Affiliations:** 1 Department of General Surgery, School of Medicine, 21 September University, Sana'a, Yemen,; 2 Department of Urology, School of Medicine, 21 September University, Sana'a, Yemen,; 3 Department of Urology, School of Medicine, Ibb University of Medical Sciences, Ibb, Yemen,; 4 Department of General Surgery, School of Medicine, Ibb University of Medical Sciences, Ibb, Yemen,; 5 Department of Internal Medicine, Faculty of Medicine, Hadhramaut University, Hadhramaut, Yemen

**Keywords:** Esophagus, papillary thyroid carcinoma, squamous cell carcinoma, synchronous neoplasm, case report

## Abstract

Papillary thyroid cancer (PTC) coexistent with esophageal squamous cell carcinoma (SCC) is of rare occurrence. We report a 45-year-old female who presented with painless anterior neck swelling for the past year. Ultrasonography showed a left hypoechoic thyroid mass measured 20x13 mm without lymph node enlargement. The fine-needle aspiration cytology was suggestive of PTC. Consequently, total thyroidectomy with bilateral neck dissection was performed. Incidentally, a small mass measuring 4x2 cm arising from the esophageal wall was identified and resected. Postoperatively, the patient developed a small esophageal fistula which was treated conservatively. The histopathological examination confirmed the diagnosis of PTC and SCC of esophageal mass. The patient underwent radiotherapy, and radioactive iodine therapy, and had acceptable conditions within two years of follow-up. In conclusion, even though the coexistence of PTC and esophageal SCC is rare, the possibility of concurrence of both tumors should be considered if an incidental mass was identified intraoperatively.

## Introduction

Papillary thyroid carcinoma (PTC) accounts for 85% of thyroid cancers, representing the most common thyroid cancer and the seventh most common cancer in females. Risk factors include, but are not limited to, ionizing radiation exposure, female gender, smoking, obesity, excess dietary iodine, alcohol, nitrates-rich diets, diabetes mellites, and other genetic factors [1]. Squamous cell carcinoma (SCC) accounts for the majority of esophageal carcinoma cases worldwide. The indolent nature of this tumor, with early-stage lymph node involvement, renders it a highly aggressive tumor with a high mortality rate. Occasionally, patients can have a metastatic SCC while completely asymptomatic [2]. There are sparse reports of PTC and esophageal SCC coexistence in the literature [3,4]. We herein report a case of a 45-year-old female patient diagnosed with PTC and esophageal SCC confirmed histopathologically; furthermore, we reviewed the literature on the clinical evaluation management of similar cases.

## Patient and observation

**Patient information:** a 45-year-old female patient presented with anterior neck swelling for the past year. The swelling was painless, with no associated dysphagia, odynophagia, weight loss, or epigastric pain. However, she noticed a rapid progression of the swelling recently. The patient was a non-smoker and has no chronic medical conditions or family history of malignancy.

**Clinical findings:** the thyroid gland appeared normal upon physical examination, and no abnormalities except a few swellings were identified on the left side.

**Diagnostic assessment:** ultrasonography (US) examinations showed a left hypoechoic thyroid mass measured 20x13 mm, located centrally and just to the left of the midline of the neck, no sizable cervical lymph nodes enlargement or other masses were detected. Using US guidance, fine-needle aspiration cytology from the left thyroid mass revealed PTC. The patient workup was unremarkable, including thyroid function tests, chest X-ray, electrocardiogram (ECG), and pulmonary function.

**Figure 1:**
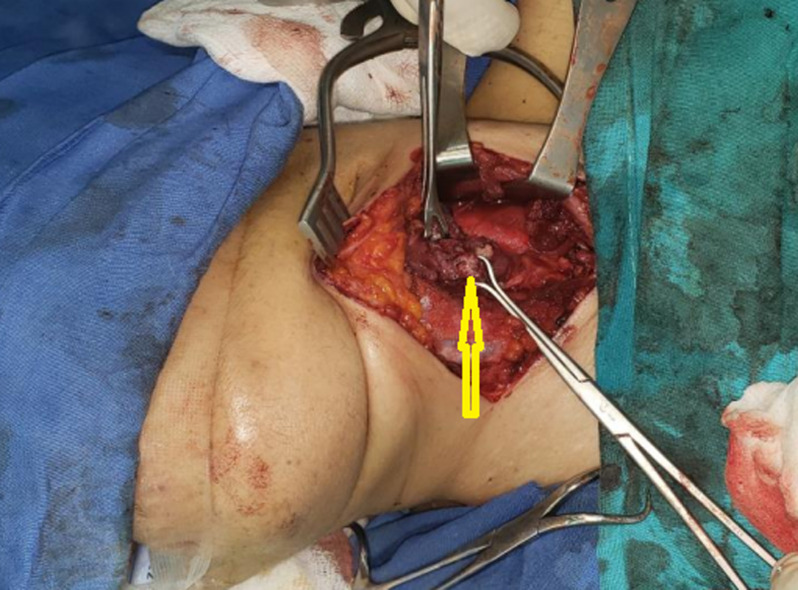
intraoperative photo showing a small mass arising from the esophagus (arrow)

**Therapeutic intervention:** the patient was diagnosed with PTC, and total thyroidectomy with bilateral neck lymph node dissection was performed subsequently. Intraoperatively, there was no significant lymph node enlargement on any side of the neck. Instead, we found about 4x2 cm mass on the left side of the neck-deep to the trachea; arising from the esophageal wall, with the assistance of a nasogastric tube (NG tube), local excision of the mass was done, and the remaining of esophageal wall was closed over the NG tube (Figure 1).

**Figure 2:**
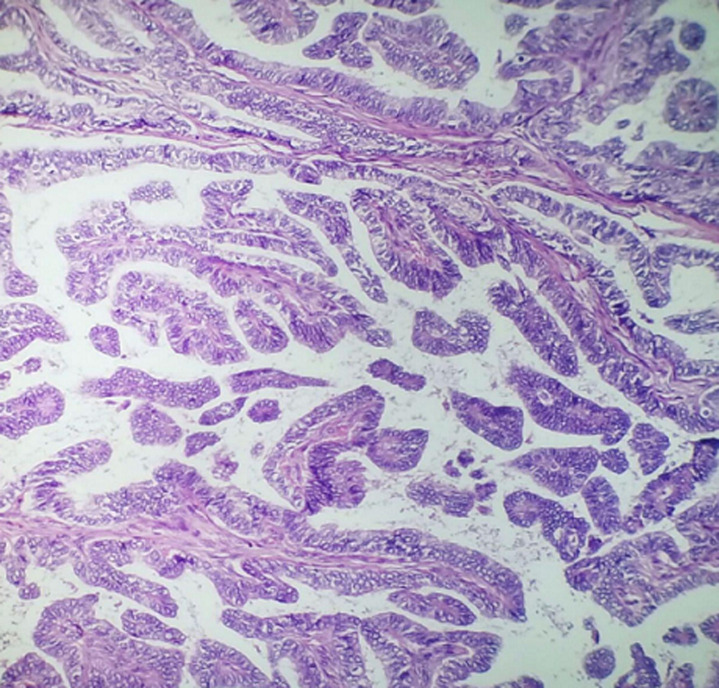
histopathologic examination of thyroid mass consists with papillary thyroid carcinoma

**Figure 3:**
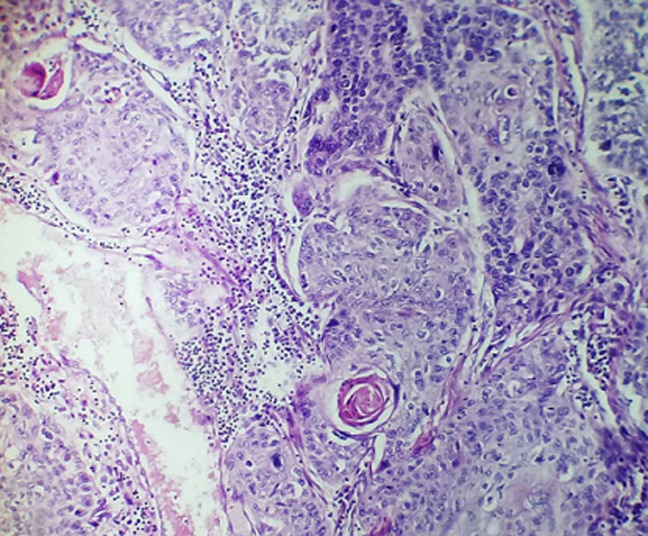
histopathologic examination of resected mass revealed a well-differentiated squamous cell carcinoma of the esophagus

**Follow-up and outcome:** postoperatively, the patient developed a small esophageal fistula on the seventh day, which was treated conservatively for 8 weeks (NG tube feeding, complete High protein diet via NG tube, hyperalimentation via intravenous route, skin care around the fistula including debridement and wound dressing, and proper antibiotic therapy). The histopathology of the resected mass revealed a stroma organized in a coarse papillary pattern, with fibro-vascular cores and oncocytic cells with nuclear grooves and intranuclear inclusions. In addition to uneven nuclear contours, nuclear enlargement, overlapping, grooves, and nuclear pseudo-inclusions were seen (Figure 2). Furthermore, the tumor cells' immunohistochemical staining showed strong positivity for thyroglobulin, indicating thyroid origin. There was no evidence of extrathyroidal extension, vascular invasion, regional lymph node involvement, or distant metastasis. The esophagectomy specimen revealed a 4x2 cm ulcerative lesion located in the distal third of the esophagus. Microscopic examination of the tumor identified a moderately differentiated SCC extending to the submucosa (Figure 3). The patient returned for further investigations, including computed tomography (CT) scan for the neck and chest with oral and IV contrast, which revealed no residual esophageal, thyroidal masses, or enlarged lymph nodes. There was small leakage of contrast from the lateral esophageal defect (10-15 mm in size), indicating a minor esophageal fistula. The patient was then referred to the National Oncology Center for complete management of both cancers by radioactive iodine therapy and radiotherapy. The patient received radioactive iodine (radioiodine) 8 weeks following the surgery, and the adjuvant external beam radiation therapy with 50 to 75 gay started at 16 weeks following the surgery. Within two years of follow-up, the patient had acceptable conditions and only complained of mild odynophagia.

**Patient perspective:** the patient was pleased with the care she received throughout therapy.

**Informed consent:** written informed consent was obtained from the patient for participation in our study.

## Discussion

The introduction of innovative diagnostic procedures and the longer life expectancy have raised the global incidence of diagnosing multiple primary malignant neoplasms in a single patient. Regardless, co-existing primary cancers of the esophagus and thyroid gland remain extremely rare [3]. The current study reports the coexistence of PTC and esophageal SCC. Reviewing the literature regarding this coexistence is rare. Similar published cases were reported by Cheng *et al*., Naomoto *et al*., Juhász *et al*., Resta *et al*., and Mattavelli *et al*. [3,5-8]. Telomerase reverse transcriptase (TERT) promoter mutations were recognized in various malignancies with variable frequencies. Telomerase reverse transcriptase (TERT) promoter mutations in thyroid cancer are more frequently observed in aggressive undifferentiated and anaplastic thyroid carcinomas than in PTCs. However, TERT promoter mutation incidence in solid variant PTC can be underestimated due to the rarity of these tumors and the genetic variability. Additionally, associated alterations among subvariants of PTC were limited to study [4]. Recent studies have shown a slightly higher prevalence of TERT promoter mutations in solid variant PTC than that in conventional PTC but significantly lower than that of tall cell variant PTC [4,9,10]. We did not perform any mutational analysis due to the non-availability of these tests in our country and the patient's low socioeconomic status. The presentation of thyroid cancer can be variable, with the majority of cases presented with a thyroid nodule, noticed by the patient, the clinician, or during imaging. Other symptoms may be linked to the tumor mass-effect or invasion, including dysphagia, dyspnea, hoarseness, or a change in voice, among others, as seen in our case [11]. Thyroid US and US-guided fine-needle aspiration biopsy (FNAB) has facilitated the detection of PTC. Calcification, echogenicity, and blood flow can be detected using the US. For instance, microcalcification is commonly observed in PTC and is typically absent in follicular thyroid carcinoma.

Although PTC commonly has a favorable clinical outcome with an excellent prognosis, regional lymph node and distant metastasis have been widely described in the literature [3]. In our patient, the imaging findings of the asymptomatic nodules and the absence of lymph node enlargement were highly suggestive of PTC. A computerized tomogram (CT) or magnetic resonance image (MRI) could help distinguish direct invasion from metastasis [12]. However, they have low sensitivity and are typically reserved for cases with symptoms and signs suggestive of local invasion, palpable lymph nodes on examination, or extensive lymphadenopathy in the US [13,14]. Our patient clinical presentation and US findings hinted toward a localized tumor. The management of differentiated thyroid cancer is primarily surgical, including total/near-total thyroidectomy or unilateral lobectomy with isthmectomy. The surgical approach depends on the disease extension (e.g, initial tumor size and the occurrence of extrathyroidal extension or lymph node metastases), patient´s age, and comorbidities. Generally, lobectomy is recommended for patients with an indeterminate solitary nodule, and total thyroidectomy with bilateral neck dissection is advised for patients with papillary or follicular cancer with a primary tumor >4 cm in diameter, extrathyroidal progression of the tumor, or lymph node, or distant site metastases, as performed in our patient [11].

Primary esophageal SCC with concurrent PTC is rarely reported in the literature. Classically, it presents with progressive dysphagia or retrosternal discomfort. In addition, it typically arises in patients with a history of smoking or alcohol misuse. None of each was reported in our patient. Although endoscopic US provides a highly sensitive to diagnose esophageal SCC, conventional US has minimal utility; in addition, CT scan can be helpful in diagnosing occult metastasis [3,4,15]. In our case, an esophageal mass was incidentally discovered intraoperatively and resected. This rare presentation was described by *Cheng et al*. [3]. In our case, the postoperative pathological examination revealed an esophageal SCC. Therefore, a neck CT scan was performed postoperatively and showed no remanent malignant tissue in the esophageal area. Managing advanced thyroid malignancies is extremely challenging, particularly when there is an extranodal and extracapsular tumor extension. A multidisciplinary approach is recommended with a combination of surgical intervention combined with radiotherapy would be a reasonable option in managing these tumors. Postoperative radio-ablation or external beam radiotherapy may be chosen; however, the latter is preferable when gross residual thyroid tissue is left as the required high doses of radio-iodine may result in excessive toxicity in a disease that is already destined to have a bad outcome [16]. Our patient was developed with a small fistula treated with conservative management (the patient did not accept additional surgeries). Reconstruction with a myocutaneous flap may be needed if more than 50% of pharyngeal tissue was resented [12]. However, the presence of other treatment options, including debulking surgery and adjuvant postoperative radiotherapy/radio ablation, make it unacceptable to the surgeons and for the patient because of the inherent morbidity.

## Conclusion

Even though the coexistence of PTC and esophageal SCC is of rare occurrence, the surgeon should consider the possibility of synchronous of those tumors if any incidental mass is identified during surgery.
